# Repurposing antimicrobial stewardship tools in the electronic medical record for the management of COVID-19 patients

**DOI:** 10.1017/ice.2020.281

**Published:** 2020-06-08

**Authors:** Matthew W. Davis, Dayna McManus, Alan Koff, Gregory R. Jaszczur, Maricar Malinis, Charles Dela Cruz, Clemente J. Britto, Christina Price, Veronica Azmy, Kelsey Kaman, David Gaston, Kevin Early, Michelle DeWitt, Ju-Sung Song, Claudia Ortiz, Manisha Juthani-Mehta, Jeffrey E. Topal

**Affiliations:** 1 Department of Pharmacy Services, Yale New Haven Hospital, New Haven, Connecticut; 2 Section of Infectious Diseases, Yale University School of Medicine, New Haven, Connecticut; 3 Information Technology Services, Yale New Haven Health System, New Haven, Connecticut; 4 Section of Pulmonary, Critical Care and Sleep Medicine, Yale University School of Medicine, New Haven, Connecticut; 5 Section of Rheumatology, Allergy and Immunology, Yale University School of Medicine, New Haven, Connecticut

## Abstract

During the COVID-19 pandemic, the antimicrobial stewardship module in our electronic medical record was reconfigured for the management of COVID-19 patients. This change allowed our subspecialist providers to review charts quickly to optimize potential therapy and management during the patient surge.

In December of 2019, several cases of pneumonia of unknown etiology were recognized by the World Health Organization (WHO) in Wuhan City, Hubei Province, China.^1^ This pneumonia was rapidly identified to be caused by the SARS-CoV-2 virus and named coronavirus disease 2019 (COVID-19). The presence of human-to-human transmission was quickly recognized, and on March 11, 2020, COVID-19 was declared a pandemic by the WHO.^2–5^

In the United States, the first documented case of COVID-19 occurred in a 35-year-old man in Snohomish County, Washington, on January 19, 2020.^6^ Rapid spread subsequently occurred across the country, and as of April 23, the United States had recorded >800,000 cases and >44,000 deaths.^7^

Given the spread of this novel virus, hospitals have seen a rapid increase of COVID-19 patients in a short period. This surge has placed a strain on vital resources, including personal protective equipment (PPE), mechanical ventilators, and hospital personnel, including infectious disease consultants.

At Yale New Haven Hospital (YNHH), in anticipation of a surge in COVID-19 patients, a multidisciplinary team comprised of members from allergy and immunology, antimicrobial stewardship team (AST), hematology, infectious diseases, pharmacy, pulmonology, and critical care collaborated to provide guidance on potential treatments and management. One of the primary clinical challenges to be addressed was the increasing number of new COVID-19 patients each day. At the height of the surge, in our 1,500-bed hospital, we were caring for >450 patients with COVID-19. With this rapid increase, the number of total medical ICU beds was expanded from 56 to 131. Prior to the surge, our 4 inpatient infectious diseases consultants covered ~50 patients. However, during this time, the hospital staff requested subspecialty guidance for the management of COVID-19 patients. The number of patients with COVID-19 overwhelmed our ability to provide optimal subspecialty care. Given this increase, there was a significant need for a rapid assessment tool to review patients and to prioritize consultations.

Prior to the COVID-19 pandemic, the YNHH AST customized the antimicrobial stewardship (AMS) module in our electronic medical record (EMR; Epic, Verona, WI). This customized module facilitated efficient review of relevant clinical patient information to provide recommendations for optimizing antimicrobial use. The existing AMS accordion report contained information regarding vital signs, fever curve, intake and output, relevant hematology and chemistry laboratory values, microbiology results, radiology studies, medications, and allergies. Given the rapid increase in number of patients and the need to provide clinical oversight, a challenge similar to AMS, a revised version of this tool was proposed by the AST as a potential solution.

Herein, we describe our process of repurposing the AMS module and associated technology to optimize the management of COVID-19 patients.

## Intervention

To identify COVID-19 patients in real time, with the help of the information technology (IT) team, 2 patient lists were created in the EMR. The first list comprised inpatients with positive COVID-19 results based on SARS-CoV-2 polymerase chain reaction assay (PCR). The second list identified patients who were suspected to be COVID-19 positive based on clinical presentation with a SARS-CoV-2 PCR in process. At YNHH, in-house SARS-CoV-2 PCR testing was initiated on March 13, 2020, which allowed for an initial turnaround time of 8–12 hours. Prior to this, SAR-CoV-2 tests were done through the Connecticut Department of Health. The validation of the in-house testing was timely because our first COVID-19–positive patient was admitted on March 12, 2020.

**Fig. 1.**
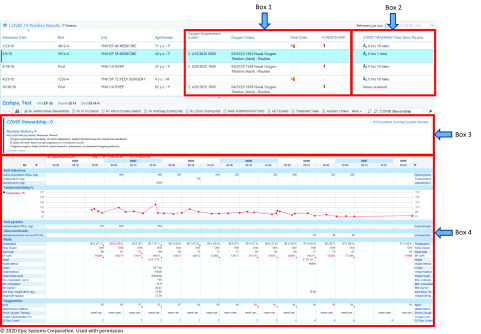
An image displaying the customized AMS module within our EMR, EPIC. Box 1 illustrates the columns added specifically for COVID-19; oxygen requirement, oxygen orders, vent order and high sensitivity CRP to triage higher acuity patients. Box 2 demonstrates the “COVID TRACKING Time Since Review” which shows the last time a patient has been reviewed to prioritize patients. Box 3 is the “Document System Scoring Report”, where documentation for follow-up items can be entered. Box 4 is part of the AMS accordion report which contains vital signs, fever curve, intake/output, relevant hematology and chemistry laboratory values, microbiology, radiology, medications and allergies. Due to space limitations, this is a snap shot of the report so not all of the available data is displayed. © 2020 Epic Systems Corporation. Used with permission.

After the creation of the COVID-19 patient lists, additional modifications were made to specifically aid in the assessment of these patients. In addition to the standard columns within the patients list (ie, admission date, bed, unit, patient name, age, and gender), the following items were added to help rapidly identify patients with more severe disease: oxygen requirements, oxygen orders, and the presence of mechanical ventilation orders (Fig. 1, box 1). To further triage higher-acuity patients, a column delineating high-sensitivity C-reactive protein (hsCRP) levels was also created. A dynamic scoring column was built which would display a designated icon if patients had an hsCRP ≥70 mg/L or an increase ≥30 mg/L in the previous 24 hours (displayed as COVID-19 CRP). HsCRP can be elevated in the setting of the cytokine release syndrome phase of COVID-19, and it is a reliable indicator of disease severity (Fig. 1, box 1).^8^ Furthermore, the AMS accordion report described earlier was modified to also include COVID-19–specific information including inflammatory markers and potential treatment options.

A “COVID TRACKING time since review” column was incorporated into the patient list to display the amount of time since the patient was last reviewed (Fig. 1, box 2). If the patient had not been reviewed, then the phrase “never reviewed” was displayed in this column. When a patient was selected from the list, the optimized AMS accordion report would display relevant COVID-19 data so that the patient could be reviewed without the need to enter data into the electronic chart (Fig. 1, box 4). Within the accordion report, a document scoring system could be used as a hand-off tool to provide a brief synopsis of important factors and items to follow up in subsequent reviews (Fig. 1, box 3). The option to post to a progress note was available within the documentation screen if a specific recommendation needed to be placed into the patient’s chart to facilitate communication with the primary team. When the review was completed, the “COVID TRACKING time since review” column was automatically updated to help avoid duplicate reviews of the same patient.

This customized AMS module became available 2 weeks from the admission our first COVID-19–positive patient, and education on this tool was provided by the AST. The multidisciplinary team members, including attending physicians and fellows, review patients with COVID-19 infection on a daily basis to support and optimize care in conjunction with the primary team.

## Discussion

As a result of the COVID-19 pandemic, consultants are faced with an unprecedented number of patients to evaluate in a short period. This significant challenge requires consultants to develop new work flows to provide optimal patient care.

Similarly, the AST is challenged with reviewing large numbers of patients, often with limited personnel. Therefore, many ASTs rely on optimizing the EMR to quickly identify and review patients. The use of technology for antimicrobial stewardship has been shown to improve the efficiency in identifying and optimizing the management of patients.^9^ With these tools, repurposing the AST workflow and AMS module at our institution allowed us to quickly adapt to the challenges the pandemic posed as our COVID-19 census rapidly exceeded 25% of our total hospital capacity. This workflow offers an efficient way for the consultant to make timely clinical assessments and recommendations including potential therapeutic options, clinical trial enrollment, anticoagulation, monitoring adverse effects from treatment, and antimicrobial use. For the complex COVID-19 cases, a formal infectious disease consultation was recommended if indicated.

In conclusion, during a pandemic such as COVID-19, with a surge in hospitalized patients, alternative mechanisms for patient review may need to be utilized. To help meet this unprecedented challenge, repurposing the existing AMS module and workflow can potentially facilitate appropriate patient review and timely intervention.
